# Lipid Profiles and *trans* Fatty Acids in Serum Phospholipids of Semi-nomadic Fulani in Northern Nigeria

**DOI:** 10.3329/jhpn.v28i2.4886

**Published:** 2010-04

**Authors:** Robert H. Glew, Lu-Te Chuang, Tammy Berry, Henry Okolie, Michael J. Crossey, Dorothy J. VanderJagt

**Affiliations:** ^1^ Department of Biochemistry and Molecular Biology, University of New Mexico School of Medicine, Albuquerque, New Mexico, USA; ^2^ Department of Biotechnology, Yuanpei University, Hsin Chu, Taiwan; ^3^ Department of Medicine, Federal Medical Centre-Gombe, Gombe, Nigeria; ^4^ TriCore Reference Laboratories, Albuquerque, New Mexico, USA

**Keywords:** Lipid profile, Fulani, Serum, *Trans* fatty acids, Nigeria

## Abstract

The Fulani are semi-nomadic pastoralists of West Africa whose diet, culture, and economy are centred on cattle. Previous studies have shown that the Fulani of northern Nigeria derive 50% of their total calories from fat and 30% of their calories from milk, cheese, yogurt, and butter oil that contain significant amounts of *trans* fatty acids (TFAs), primarily vaccenic acid, which raise total serum cholesterol and low-density lipoprotein-cholesterol (LDL-C), and lower high-density lipoprotein-cholesterol (HDL-C). The study was conducted to know how the consumption of relatively large amounts of dairy products by adult Fulani affected the TFA content of their serum phospholipids. Blood samples were collected from 22 male and 29 female Fulani, aged 35–60 years, who were living in rural areas of Gombe state in northeastern Nigeria. The total serum phospholipid fraction was isolated, and its fatty acid composition was determined. Surprisingly, vaccenic acid was not detected, and three other TFAs—18:1-t6, 18:1-t9, and 18:2-t9, t12—together accounted for only 0.16% of the total fatty acid. The mean serum total cholesterol, LDL-C, and triglyceride concentrations of the subjects were within the normal range for populations in developed countries; however, at 32 mg/dL, the mean serum HDL-C concentration of the Fulani males was slightly below the lower limit of the reference range. No correlations were observed between the total TFA percentage or that of the three individual TFAs and any of the parameters of the serum lipid profile. These findings indicate that, with respect to TFAs at least, the fatty acid pattern of the serum phospholipids of Fulani pastoralists does not reflect the high TFA content of their traditional diet. Despite the consumption of rumenic acid-rich dairy products, for unknown reasons, the semi-nomadic Fulani manage to maintain a low level of TFAs in their blood and a relatively healthful serum lipid profile. While the mechanism that accounts for this disconnect between the consumption of TFAs by Fulani pastoralists and the proportion of TFAs in their serum phospholipids is obscure, possibilities include discrimination against rumenic acid during the process of triglyceride synthesis and chylomicron synthesis in the intestine and the preferential oxidation of TFAs by Fulani the people compared to other ethnic groups.

## INTRODUCTION

It is widely accepted that consumption of *trans* fatty acids (TFAs) increases the risk of coronary heart disease: these promote inflammation and increase the levels of markers of inflammation, e.g. tumor necrosis factor-α, interleukin-6, and C-reactive protein (1, 2). TFAs also raise plasma total cholesterol (TC), low-density lipoprotein-cholesterol (LDL-C), and triglycerides (TG), which are risk factors of cardiovascular diseases (CVDs), while these lower the level of cardioprotective HDL-C in plasma (3).

We learned from past studies on diet and CVD risk factors of the semi-nomadic Fulani people of northern Nigeria whose culture and economy are centred on cattle that, despite deriving approximately 30% of their total calories from dairy products rich in saturated fatty acids, Fulani men and women had serum levels of TC, LDL-C, and TG that were within normal reference ranges for Western populations (4, 5). A subsequent study on the fatty acid composition of the serum phospholipids of Fulani adults revealed a high proportion of saturated fatty acids compared to non-Fulani ethnic groups in southern Nigeria and populations in Europe, the United States, and Australia; however, TFAs were not determined in that 2003 study (6).

The issue of TFA levels in Fulani pastoralists is of interest since dairy products contribute substantially to their diet and because the milk and butter oil they derive from their cows and which they consume regularly contain 4.6% and 10.4% *trans* isomers of oleic acid respectively (7). The main TFA in ruminant fats is 11-*trans*-18:1 isomer (11*t*-18:1; vaccenic acid) (8) whereas partially-hydrogenated (9) vegetable oils mainly have the 9*t*:18:1 isomer (elaidic acid) (10). Based on two previous studies by us (4, 5), we estimate that TFAs account for 1.0–1.5% of the total energy intake of rural Fulani people in northern Nigeria, which is a level of consumption that has been shown to significantly increase the risk of coronary heart disease (3). Since dietary intake of TFAs correlates positively with blood levels of TFAs (10–12), we would, therefore, expect to find relatively high levels of TFAs in the serum phospholipids of Fulani adults. On the other hand, the favourable serum lipid profiles we have documented in this semi-nomadic population (4, 5) would seem to be inconsistent with a high proportion of TFAs in their serum phospholipids.

We, therefore, determined the fatty acid composition of the total serum phospholipid fraction of 29 Fulani women and 22 Fulani men living in the vicinity of Gombe in northeastern Nigeria. Since the fatty acid composition of human plasma phospholipids reflects recent dietary fatty acid intake (10–12), we hypothesized that, in light of the fact that TFA-rich dairy products are a major component of the diet of Fulani pastoralists (5, 6), their serum phospholipids should contain appreciable proportions of TFA. Since we were also interested in possible correlations between the TFA and the body-composition characteristics of the Fulani adults, we used bioelectrical impedance to estimate the body fat and lean body mass of the subjects in the present study.

## MATERIALS AND METHODS

### Study subjects

Healthy Fulani (Fulbe) men (n=22) and women (n=29) aged over 18 years were recruited consecutively over a five-day period from among relatives accompanying patients to the General Medicine Clinic at the Federal Medical Centre-Gombe in Gombe. Gombe, the capital of Gombe state, is located in northeastern Nigeria and has a population of approximately 270,000. The major occupations of the Fulani people in the region are farming and rearing of livestocks. The exclusion criteria included use of alcohol or tobacco. Information on age, weight, and height was recorded. A fasting blood sample was obtained for the analysis of serum lipids.

### Anthropometric measurements

A stadiometer accurate to 0.25 cm and a battery-operated scale (model number SAB700DQ-01, Sunbeam, Inc., Boca Raton, FL, USA) accurate to 0.2 kg were used for determineing, respectively, the height and weight of each subject. Blood pressure was measured using a nylon cuff and latex inflation system (Prestige Medical, Inc., Northridge, CA, USA). Fat-free mass (FFM) and body fat (BF) were estimated by bioelectrical impedance analysis conducted using a portable analyzer (Quantum, RJL Systems, Clinton Township, MI, USA) as described elsewhere (13–16). Reactance and resistance values, together with age, gender, height, and weight, were used for calculating FFM, BF, and phase angle using the software provided by the manufacturer.

### Analysis of serum lipids

Fasting blood samples obtained by venipuncture were allowed to clot for 45 minutes and then centrifuged to separate the serum fraction. The sera were aliquoted into cryovials and stored at −40 °C for four weeks until they were transported in the frozen state to Albuquerque, NM, USA, for analysis. Total cholesterol was determined by the end-point colorimetric method of Allain *et al*. (17) using a Vitros 950 analyzer. HDL-C was determined using Kodak Vitros cholesterol slides and a Vitros 250 analyzer. LDL-C was determined directly using an immunologic method (18). The lipid profile was determined in a clinical chemistry laboratory accredited by the College of American Pathologists.

### Analysis of fatty acids

Total serum lipids were extracted using a minor modification of the Folch method (19). Briefly, 1 mL of serum was extracted with 20 mL of chloroform/methanol (2:1, v/v) at 4 °C for 18 hours. The extracted lipids in the chloroform phase were separated from the aqueous phase by addition of 4 mL of 0.9% (w/v) NaCl solution. The lipid-containing chloroform phase was evaporated under a stream of dry nitrogen gas. To isolate the phospholipids, the total lipid fraction was subjected to thin-layer chromatography using hexane/diethyl ether/acetic acid (70:30:1, v/v/v) as the developing solvent. The total phospholipid fraction was scrapped from the plate and treated with 2 mL of 1% (v/v) sulphuric acid in methanol and 0.5 mL of dimethyl sulphoxide for 20 minutes at 95 °C to generate fatty acid methyl esters (FAMEs) (20). FAMEs were extracted into hexane and separated and quantified by gas chromatography using an Agilent 6890 gas chromatograph equipped with a flame ionization detector and a fused-silica capillary column (HP-88; 100 m × 0.25 mm inside diameter × 0.2 μm film thickness) (Hewlett-Packard, Sunnyvale, CA, USA). The operating conditions were as follows: helium was used as the carrier gas; the injector temperature was 250 °C and that of the detector was 280 °C; the oven temperature was set initially at 175 °C, raised to 220 °C at a rate of 2 °C per minute; held at 220 °C for 10 minutes, raised to 240 °C at a rate of 2 °C per minute; and finally held at 240 °C for five minutes. The fatty acid peaks were identified by comparing their retention times with those of known standards. For quantization of FAMEs, a known amount of the internal standard triheptadecanoin was added to each sample. The FAME standards were purchased from the Nu-Chek-Prep, Inc. (Elysian, MN, USA) and included mixture RL-461 and FAME of the following fatty acids: myristelaidate (C14:1-*t*9), palmitelaidate (C16:1-*t*9), petroselaidate (18:1-*t*6), elaidate (C18:1-*t*9), vaccenate (C18:1-*t*11), conjugated linoleate (CLA), linoelaidate (C18:2-*t*9, *t*12), and eicosenoate (C20:1-*t*11). The coefficient of variation for the various fatty acid values ranged from 1.5% to 5%.

### Statistical analyses

Descriptive statistics, group comparisons, and correlations were performed using the Number Cruncher statistical software (Version 2001) (NCSS, Kaysville, UT, USA). Since body-composition parameters and serum lipid concentrations are known to be gender-dependent, data for male and female subjects were analyzed separately. A p value of 0.05 was considered significant.

### Ethics

The Ethics Committee of the Federal Medical Centre-Gombe and the Human Research Review Committee of the University of New Mexico Health Sciences Center, Albuquerque, NM, USA, approved the study. Informed consent was obtained from each subject after the purpose and requirements of the study were explained in English or Hausa, the predominant language used in the area.

## RESULTS

### Characteristics of study population

The study population included 22 males aged 36–76 (mean 55.5) years, and 29 women aged 35–60 (mean 47.6) years (Table 1). The lean nature of the men and women that was evident in their relatively low body mass index (BMI) values (21.6 and 21.1 kg/m^2^ respectively) was confirmed by the results of bioelectrical impedance analysis of their body composition: men had 13.8% fat, and women had 26.5% fat. Although the mean (systolic/diastolic) blood pressure of both male (137/84 mmHg) and female subjects (137/82 mmHg) indicated borderline hypertension, the mean phase angle of both the sets of subjects were in the range of values reported for individuals whose overall health is good (5, 21, 22).

**Table 1.** Summary of anthropometric characteristics of Fulani adults

Parameter	Men (n=22) Mean±SD	Women (n=29) Mean±SD
Age (years)	55.5±13.5	47.6±8.3
Height (cm)	167.0±5.6	160.2±5.2
Weight (kg)	60.3±8.1	54.2±9.9
BMI (kg/m2)	21.6±3.1	21.1±3.2
Fat (kg)	8.7±4.6	15.0±6.9
Fat (%)	13.8±5.6	26.5±7.4
FFM (kg)	51.6±4.5	39.2±3.9
FFM (%)	85.9±6.4	73.5±7.4
Phase angle (degrees)	5.7±0.71	5.4±0.82
BP systolic (mmHg)	137±19	137±18
BP diastolic (mmHg)	84±10	82±12

BMI=Body mass index;

FFM=Fat-free mass;

SD=Standard deviation

The concentration of total cholesterol was higher in women than in men (156 vs 122 mg/dL, p<0.001 (Table 2). The same was true for the LDL-C concentration: women 95 mg/dL; men 73 mg/dL (p=0.005). Conversely, the female subjects had a higher serum HDL-C concentration (41 vs 32 mg/dL, p=0.007). The serum triglyceride level was slightly higher in women than in men (100 vs 91 mg/dL) but the difference was not significant. Taken together, except for the relatively-low HDL-C values for both male and female subjects, the lipid profiles of the Fulani adults in the present study would be considered within the normal reference ranges set by most developed countries (23, 24).

**Table 2.** Summary of lipid profiles of Fulani adults

Lipid profile	Men (n=22) Mean±SD	Women (n=29) Mean±SD	p value
Total cholesterol (mg/dL)	122±25	156±32	<0.001
HDL-cholesterol (mg/dL)	32±7	41±13	0.007
LDL-cholesterol (mg/dL)	73±22	95±29	0.005
Triglycerides (mg/dL)	91±35	100±45	NS

HDL=High-density lipoprotein;

LDL=Low-density lipoprotein;

NS=Not significant (p=<0.05);

SD=Standard deviation

### Fatty acid composition of serum phospholipids

Table 3 is a summary of the percentages of 26 fatty acids we analyzed in the total phospholipid fraction of the sera of the male and female Fulani subjects in the present study. Vaccenic acid (18:1-*t*11) was not detected. The *t*-6 and *t*-9 isomers of oleic acid accounted for approximately two-thirds of the total TFAs whereas the di-unsaturated TFA linolaidic acid (18:2-*t*9, *t*12) accounted for the other one-third. The most common conjugated linoleic acid present in cow's milk–rumenic acid (9-*cis*, 11-*trans* linoleic acid) was not detected in the serum phospholipids of either the male or female subjects. Significant differences in the proportions of individual fatty acids in the serum phospholipids of the male and female subjects were not observed, except for *cis*-vaccenic acid (18:1n-7) and arachidic acid (20:1) (Table 3). Collectively, the three TFAs accounted for only 0.15–0.16% of the fatty acid total.

**Table 3.** Fatty acid composition of serum phospholipids of Fulani adults

Fatty acid	Males (n=22)	Females (n=29)	All subjects (n=51)
	Mass percentage of fatty acids (mean±1 standard deviation)		
14:0 (myristic acid)	0.24±0.09	0.20±0.10	0.22±0.10
15:0 (pentadecanoic acid)	0.16±0.03	0.13±0.05	0.14±0.04
16:0 (palimitic acid)	30.1±1.95	30.2±1.86	30.2±1.88
16:1n-7 (palmitoleic acid)	0.45±0.15	0.39±0.16	0.41±0.16
18:0 (stearic acid)	12.4±1.43	13.2±1.99	12.9±1.81
18:1n-9 (oleic acid)	10.4±1.36	9.70±1.13	10.0±1.27
18:1n-7 (cis-vaccenic acid)	1.23±0.30	1.00±0.19	1.09±0.26
18:1-trans*	0.12±0.05	0.10±0.04	0.11±0.05
18:2n-6 (linoleic acid)	20.0±1.74	19.8±2.03	19.9±1.90
18:2-trans (trans-linonelaidic acid)	0.04±0.01	0.05±0.04	0.04±0.04
18:3n-6 (γ-linolenic acid)	0.14±0.07	0.14±0.07	0.14±0.07
18:3n-3 (α-linolenic acid)	0.19±0.05	0.17±0.04	0.18±0.05
20:0 (arachidic acid)	0.23±0.16	0.14±0.03	0.18±0.11
20:1 (gondoic acid)	0.18±0.07	0.17±0.04	0.17±0.05
20:2n-6 (eicosadienoic acid)	0.40±0.12	0.37±0.09	0.38±0.10
20:3n-6 (dihomo-γ-linolenic acid)	3.31±0.72	3.64±0.09	3.51±0.70
20:4n-6 (arachidonic acid)	14.0±2.62	14.2±1.89	14.1±2.19
20:5n-3 (eicosapentaenoic acid)	0.44±0.27	0.41±0.17	0.42±0.21
22:0 (behenic acid)	ND	ND	ND
22:1 (erucic acid)	0.52±0.48	0.43±0.37	0.46±0.41
22:4n-6 (adrenic acid)	0.86±0.21	0.75±0.21	0.79±0.22
22:5n-6 (n-6 docosapentaenoic acid)	0.62±0.20	0.63±0.17	0.63±0.18
22:5n-3 (n-3 docosapentaenoic acid)	0.94±0.23	0.90±0.23	0.92±0.23
24:0 (lignoceric acid)	ND	ND	ND
22:6n-3 (docosahexaenoic acid)	3.06±1.27	3.19±0.94	3.14±1.07
24:1 (nervonic acid)	0.54±0.26	0.37±0.22	0.43±0.24
Total TFA‡	0.16± 0.06	0.15±0.08	0.15±0.07

*18:1-trans=18:1-t6 (petroseladic acid) plus 18:1-t9 (elaidic acid);

‡TFA=*trans* fatty acid;

ND=Not detected.

There were no statistically significant differencs in the mass percentage of fatty acids in the serum phospholipids between males and females, except for cis-vaccenic acid (18:1n-7) and arachidic acid (20:0), p=0.003 and p=0.002 respectively

The percentages of the two essential fatty acids—linoleic acid and α-linolenic acid—were 19.9% and 0.18% respectively. The proportions of two other nutritionally-important polyunsaturated fatty acids—arachidonic acid (14.1%) and docosahexaenoic acid (3.14%)—in the serum phospholipids of the Fulani adults in the present study were similar to those we reported in an earlier study of Fulani adults in northern Nigeria (25) and by other investigators elsewhere (26–29).

### Correlations between serum phospholipids fatty acids and serum lipids

We were interested in knowing if there were relationships between the proportion of TFAs in the serum phospholipids of the subjects in our study and the serum levels of total cholesterol and certain lipoproteins that are risk factors for cardiovascular diseases. When adjusted for age and gender, no significant correlations were observed among the total percentage of the three TFAs, the percentage of 18:1-*t*6 plus 18:1-*t*9, or the percentage of 18:2-*t*9, *t*12 versus total cholesterol—HDL-C, LDL-C, or TG—in either the male or female subjects. No correlations were observed between any of the anthropometric parameters with TFAs or elements of the lipid profile.

## DISCUSSION

Since we have shown that the adult Fulani men and women who inhabit northern Nigeria derive nearly 30% of their dietary calories from cow's milk, butter oil, and dairy products derived there from (4, 5) and since both milk and butter oil from the cows of the Fulani contain relatively large amounts of TFAs, most of which is vaccenic acid (18:1-*t*11) (7, 8), we expected to find at least moderate amounts of vaccenic acid in the phospholipid fraction of the sera of these pastoralists. Contrary to expectation, the main result of the present study was the finding of low levels of three TFAs but no detectable vaccenic acid in the serum phospholipids of the Fulani adults living a pastoral existence in rural northern Nigeria. The more recent data, presented in Table 3, show that the sum of the proportions of 18:1-*t*6, 18:1-*t*9, and 18:-*t*9, *t*12 in the serum phospholipid fraction of the Fulani men and women ranged from 0.15% to 0.16%, which is much below the total TFA percentages reported by other investigators for the serum phospholipids of adults elsewhere (3, 30–33).

It has been well-documented in numerous studies in different parts of the world that the TC and LDL-C levels of an individual correlate positively with the weight percentage of TFAs in his or her serum phospholipid fraction and inversely with the HDL-C level. Although the TFA proportions in the serum phospholipids of the Fulani adults in our study were low compared to those of other populations worldwide, we were, nevertheless, still interested in inquiring if there were relationship between the TFA content of the phospholipids of the Fulani men and women and the various components of their plasma lipid profiles. In fact, no such correlations were observed. It is puzzling why TFAs would be so under-represented in the fatty acid profile of the serum phospholipids of the Fulani pastoralists whose culture, economy, and diet are centred on cattle and dairy products.

Since few studies have addressed the question of possible differences in the synthesis and catabolism of TFAs between ethnic groups and populations, we can only speculate that one or more steps in the metabolism of dietary TFAs by the relatively homogenous Fulani might differ compared to fatty acid pathways of other ethnic groups and account our finding of unexpectedly low levels of TFAs in the serum phospholipids of the Fulani pastoralists. Candidate enzymes and metabolic processes that could conceivably play a role in accounting for the finding of low levels of TFAs in the serum phospholipids of the Fulani include: chylomicron synthesis and secretion in the intestine, the specificity of lipoprotein lipase, the specificity of transporters that facilitate the uptake of fatty acids across the plasma membrane of cells, the activity of acyltransferases involved in synthesis of triglycerides, and the rate at which TFAs are oxidized by the various beta-oxidation pathways. Perhaps, the Fulani people absorb TFAs poorly from the intestine and those TFAs that are absorbed are rapidly taken up by cells and either preferentially oxidized or stored as triglycerides.

A fourth possibility has to do with the unusually low (estimated) fluidity of the serum phospholipids of the Fulani. In a previous study of the Fulani population of northern Nigeria (6), we found that the mean melting point of the fatty acyl chains of the serum phospholipids of the Fulani was considerably higher than that of many other populations. In addition, the double-bond index of the serum phospholipids of the Fulani population was remarkably low. Could it be that the low fluid character of their serum phospholipids has a negative impact on the incorporation of TFAs into newly-synthesized phospholipids, thereby resulting in a low percentage of TFAs in the serum phospholipid fraction?

The results of the present study shed light on the question of why the milk-fat of the Fulani women contains so little TFAs. Our recent study that included 41 lactating Fulani women in northern Nigeria (34) found that they produced a milk-fat that contained 7–10 times less vaccenic acid and total TFA than the milk of women in France and several other developed countries. Since the mammary gland uses fatty acids contained in plasma lipoproteins to synthesize milk-fat, it is reasonable to expect that nursing Fulani women whose bodies have a low TFA content would produce a milk that contains proportionately low amounts of TFAs.

Our experience over the past 15 years in northern Nigeria informs us that the diets of the rural Fulani people in Plateau state and Gombe state where our studies were conducted tend to vary little from year to year. Nevertheless, a major limitation of the present study was that, since we did not analyze the diets of the Fulani subjects, we can only assume that their diets were similar to those of the Fulani men and women who participated in our previous studies in which dietary analysis was performed (4, 5). The Fulani adults in northern Nigeria consume a low-calorie diet (approximately 1,750 kcal per day) in which protein and fat account for 20% and 50% of calories respectively (4, 5). Milk and other dairy products, e.g. butter oil, yogurt, and cheese, supply about 30% of calories. Meat and milk together provide about 75% of total protein.

If dairy products were not a significant component of the diets of the present study subjects, our main finding of low percentages of TFAs in the serum of the Fulani adults would be expected. Motard-Belanger *et al.* and Chardigny *et al.* recently reported that a daily intake of 10–12 g of ruminant TFAs is required to raise the plasma total cholesterol and LDL-C concentrations, which far exceeds our estimates of the amount of TFAs consumed by the Fulani pastoralists in northern Nigeria (35, 36). If the actual intake of TFAs by the Fulani subjects in the present study did not exceed the range of 1–1.5 g per day we estimated based on our previous study of the diets of this same population of Fulani (4), the current finding of generally favourable lipid profiles in the Fulani men and women should not be regarded as surprising. Another limitation of our study is that the literature does not provide information regarding the incidence of cardiovascular diseases among the Fulani or statistics about the causes of death in this ethnic group.

In terms of both body composition and plasma lipid profiles, the 51 Fulani adults in the present study were similar to the 79 adults we had studied several years ago (4). Considering the information obtained in the present study, together with previous ones, we have conducted among the Fulani population of northern Nigeria, we offer at least three reasons why the lipid profiles of the Fulani men and women are generally favourable compared to norms set by most countries in Europe and North America. First, the percentage of TFAs in serum phospholipids is unusually low (Table 3). Second, although the percentage of fat in their diets and their intake of dairy products are relatively high, because the average caloric intake of the Fulani men and women is less than 2,000 kcal per day (4, 5), their absolute fat intake is only about half that of adults in most developed countries. Third, the activity level of these pastoralists is high: the men travel considerable distances with their cattle in search of water and pasture while the women commonly walk several miles each day for gathering firewood and collecting water, and do other physically-demanding chores.

According to Willet and Mozaffarian, the epidemio-logic evidence indicates that the amounts of TFAs from natural (i.e. ruminant) sources contained in western diets do not contribute importantly to the risk of coronary heart disease (37). Our findings lead us to extend their conclusion to include the semi-nomadic Fulani pastoralists of northern Nigeria whose diet includes significant quantities of dairy products.

Future studies should be aimed at assessing the metabolic machinery of the Fulani population, particularly their ability to incorporate dietary TFAs into chylomicrons and to clear TFAs from the blood circulation. Comparative studies of the ability of Fulani and other ethnic groups to synthesize triglycerides and oxidize TFAs should also be encouraged.

## ACKNOWLEDGEMENTS

This study was supported by a Minority International Research Training grant from the National Institutes of Health, USA.
